# mRNA Sequencing to Identify Aberrant Splicing in X-linked Alport Syndrome

**DOI:** 10.1016/j.ekir.2026.106553

**Published:** 2026-04-22

**Authors:** Dipti Rao, Bartholomeus T. van den Berge, Anneke T. Vulto-van Silfhout, Ilse M. Rood, Joanna A.E. van Wijk, Arend Bökenkamp, Layla Damen, Patrick Rump, Jasper J. van der Smagt, Jitske Jansen, Bart Smeets, Jack F. Wetzels, Rutger J. Maas, Michel van Geel

**Affiliations:** 1 Department of Nephrology, Radboud University Medical Center, Nijmegen, The Netherlands; 2 Department of Pathology, Radboud University Medical Center, Nijmegen, The Netherlands; 3 Department of Clinical Genetics, Maastricht University Medical Center, Maastricht, The Netherlands; 4 Department of Pediatric Nephrology, Amalia Children’s Hospital, Radboud University Medical Center, Nijmegen, The Netherlands; 5 Department of Pediatric Nephrology, Emma Children's Hospital, Amsterdam University Medical Centers, Amsterdam, The Netherlands; 6 Department of Human Genetics, Amsterdam UMC location University of Amsterdam, Amsterdam, The Netherlands; 7 Department of Genetics, University Medical Center Groningen, University of Groningen, Groningen, The Netherlands; 8 Department of Genetics, University Medical Center Utrecht, Utrecht, Netherlands; 9 Department of Medicine 2, Institute of Experimental Medicine and Systems Biology, RWTH Aachen University Hospital, Aachen, Germany

**Keywords:** genetic disease, Alport syndrome, *COL4A5* gene, intronic variants

## Abstract

**Introduction:**

X-linked Alport syndrome (XLAS) is a well-known monogenetic kidney disease caused by pathogenic variants in the *COL4A5* gene. Routine analysis of exons and direct flanking regions fails to identify a pathogenic variant in 10% to 20% of patients with XLAS.

**Methods:**

We evaluated 11 selected patients with clinical features of XLAS, in whom routine analysis failed to identify a pathogenic variant. In 2 patients a variant of unknown significance was detected in the intronic splice site regions. We used mRNA analysis from fibroblasts or urine-derived podocyte-lineage cells to establish a genetic diagnosis.

**Results:**

In 2 patients with a variant of unknown significance (VUS), mRNA analysis confirmed the pathogenicity. In 9 patients, mRNA analysis was used to evaluate aberrant splicing and guide genomic DNA sequencing. In 7 patients a novel pathogenic deep-intronic variant was found. Overall, aberrant splicing was complete in 5 patients and partial in 4, whereas kidney disease was less severe in the latter group.

**Conclusion:**

This report highlights the importance of mRNA analysis to confirm pathogenicity or facilitate the search for intronic variants to establish a genetic diagnosis in XLAS. This analysis can serve as a diagnostic tool in patients suspected for Alport syndrome (AS) when routine genetic analysis fails to identify a pathogenic variant.

## Graphical abstract

**Figure:**
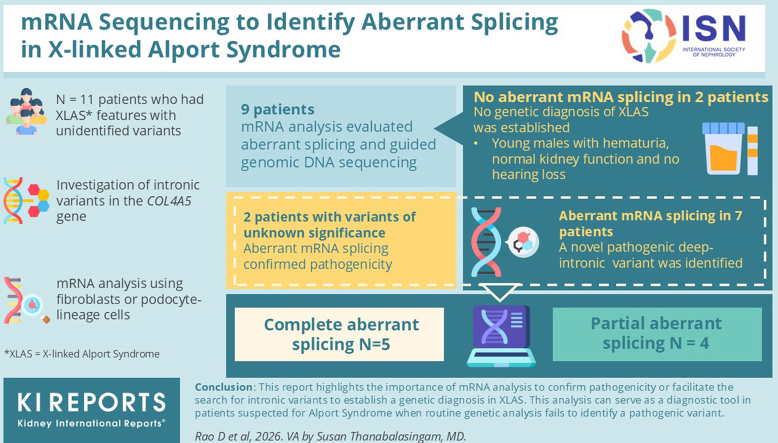

AS is a well-known monogenetic kidney disease. AS may be caused by pathogenic variants in the genes *COL4A3*, *COL4A4,* and *COL4A5*, generating defects in type IV collagen that constitute the glomerular basement membrane (GBM). The GBM is a key component of the renal filtration barrier. Absence or disruption of the collagen a3a4a5 (IV) network in the GBM underlies the renal phenotype of AS.

XLAS is caused by variants in the *COL4A5* gene, encoding for the type IV collagen-α5 chain. *COL4A5* pathogenic variants lead to aberrant or decreased expression of the collagen-α5 chain in the GBM and Bowman’s capsule in the kidneys. The collagen-α5 chain is also present in the cochlear basement membrane and base of the ocular lens; therefore, chain defects may also lead to sensorineural hearing loss and ocular lesions. GBM defects in AS initially cause hematuria, followed by proteinuria, and a decline in kidney function as the disease progresses over the years. Because of its location on the X-chromosome, male patients have a higher risk of developing end-stage kidney disease (ESKD) than female patients.^1^, ^2^, ^3^ In female patients, the phenotype is quite heterogeneous, possibly because of random X-inactivation and skewing.^4^

In patients with suspected AS, genetic testing of all 3 *COL4A3/A4/A5* genes is recommended. A genetic diagnosis is important for establishing the mode of inheritance, calculating the risk for offspring, discussing reproductive options, counseling family members, and evaluating possible kidney donors. Moreover, identifying or confirming the disease-causing variant has therapeutic consequences. It enables early initiation of renin-angiotensin-aldosterone system blockade, thus attenuating kidney function deterioration and ultimately delaying the onset of ESKD. Furthermore, establishing a genetic diagnosis is also important for novel (gene) therapy in the future.^5^, ^6^, ^7^, ^8^

Currently, the most efficient and cost-effective diagnostic test is through next-generation sequencing of exons and flanking regions combined with multiplex ligation-dependent probe amplification (MLPA). However, in 10% to 20% of patients, a causal variant cannot be detected. In some patients, variants are detected, but their pathogenicity is unknown (VUS). Since these sequencing technologies are designed to enrich for exons and immediate flanking splice sites, deep-intronic (outside the flanking regions) variants will be missed.^9^, ^10^, ^11^, ^12^, ^13^

In addition to exon sequencing of the *COL4A5* gene, immunohistochemical staining of the collagen-α5 chain in a kidney or skin biopsy is also common practice as a diagnostic test for XLAS. An abnormal staining pattern (absent or focal, segmental staining) is very suggestive of XLAS.^11^^,^^14^

Potential splicing changes of an identified VUS can be predicted by online prediction tools such as the Human Splicing Finder, MaxEntScan, and SpliceAI.^15^ However, in-silico splicing predictions require confirmation on mRNA isolated from tissue to verify the pathogenic effect of the variant.^11^^,^^16^^,^^17^ Thus, analysis of *COL4A5* mRNA is required to confirm pathogenicity of a VUS. In addition, mRNA analysis and the identification of aberrant splicing may also guide the search for variants in the deep-intronic regions not included in routine DNA testing.^7^^,^^8^

Analysis of mRNA isolated from affected kidney tissue is the most reliable method to identify splicing aberrations. Obtaining affected tissue by kidney biopsy is usually too invasive. Alternatively, mRNA can be isolated from hair follicles, cultured fibroblasts, and peripheral blood leukocytes. Tissue-specific variation in the expression of aberrant transcripts has been reported.^11^^,^^12^^,^^17^, ^18^, ^19^, ^20^, ^21^, ^22^, ^23^ Since *COL4A5* expression in hair follicles and fibroblasts is more abundant, it is preferred over peripheral blood mononuclear cells.

So far, there have been a limited number of reports successfully identifying deep-intronic variants causing splicing aberrations by analysis of mRNA. Recently, Boisson *et al.*^12^ published a cohort in which they identified deep-intronic variants in 17 out of 19 patients of XLAS using targeted RNAseq on mRNA from cultured fibroblasts. In addition, mRNA analysis directly from urine or of urine-derived podocyte-lineage cells was recently described as a noninvasive method to identify splicing aberrations in patients with genetically challenging diagnoses of AS.^24^, ^25^, ^26^

In this report, we describe 11 probands of families with clinical suspicion of XLAS and inconclusive routine genetic analysis of at least the exons of the *COL4A5* gene. We used analysis of mRNA isolated from fibroblasts (*n* = 11) or urine-derived podocyte-lineage cells (*n* = 1). In 2 patients with an identified VUS, mRNA analysis confirmed pathogenicity. In 7 patients, mRNA analysis facilitated the identification of novel pathogenic intronic variants through subsequent targeted genomic DNA sequencing.

## Methods

### Subjects

This study investigated intronic variants in the *COL4A5* gene by using mRNA analysis from fibroblasts or urine-derived podocyte-lineage cells.

Between 2006 and 2025, patients with clinical features of XLAS, with negative *COL4A5* staining, in whom routine genetic analysis of at least the exons and direct flanking regions of the *COL4A3/A4/A5* genes at the laboratory of clinical genetics at the Maastricht University Medical Center+ in the Netherlands failed to identify a pathogenic variant, were invited to undergo further evaluation, including a skin biopsy to allow culture of fibroblasts.

**Figure 1:**
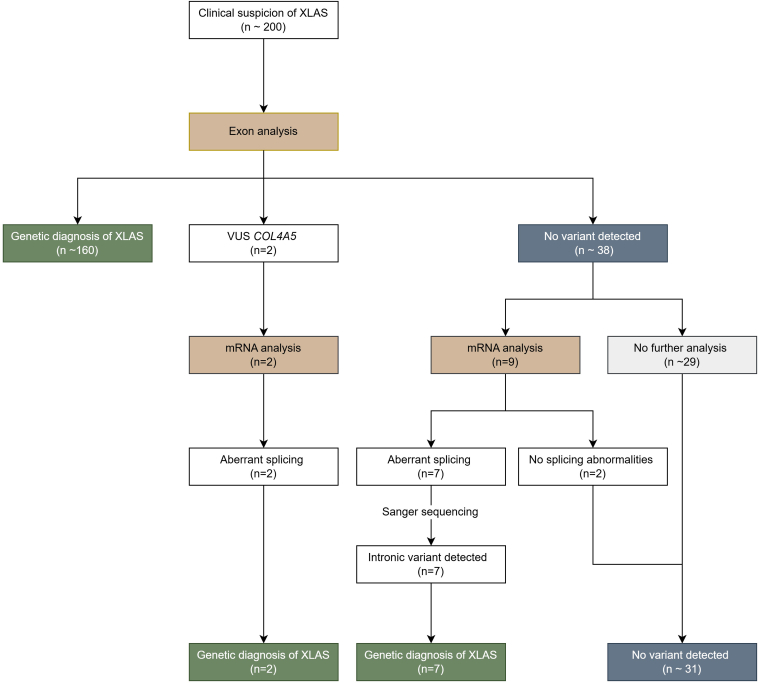
Flowchart of patient selection and estimated diagnostic yield in suspected XLAS. Based on our estimates, over a 20-year period (2006–2025), approximately 200 patients received a genetically confirmed diagnosis of XLAS through our laboratory (10/yr). Routine genetic testing is estimated to be negative in 10%–20% of patients with XLAS, corresponding to ∼ 40 patients, and a confirmed genetic diagnosis in ∼ 160 patients. This study focuses on further evaluation of patients with negative COL4A5 staining, who did consent to culture of fibroblasts for further evaluation and consented for this study. Using mRNA analysis, a genetic diagnosis was confirmed in 2 patients with a VUS in COL4A5 and 7 out of 9 patients with negative routine genetic analysis. There is no information on those who did not consent to further analysis. These data suggest that the likelihood of establishing a genetic diagnosis using mRNA analysis in patients with suspected XLAS and initially negative genetic testing ranges from approximately 20% (7 of ∼ 40, assuming negative results in the remaining ∼ 29 patients) to 77% (7 of 9). The lower estimate likely underestimates the true diagnostic yield, as clinical suspicion of XLAS was probably lower in patients who did not undergo further testing. VUS, variant of unknown significance; XLAS, X-linked Alport syndrome.

Eleven patients returned to the hospital and provided consent (Figure 1). In those patients, we performed mRNA analysis, either for confirmation of pathogenicity of an identified VUS (*n* = 2) or for screening of aberrant splicing (*n* = 9). Clinical data were obtained from the medical records of the participants with a detected variant. Upon inclusion, the family history was updated. All participants provided informed consent for the study, which was approved by the ethical committee (Maastricht UMC+ METC 2022-3204).

### Definitions

XLAS was defined based on clinical criteria including hematuria, proteinuria, and progressive decline in estimated glomerular filtration rate, in combination with histopathological criteria, specifically lamellated GBM, and/or abnormal collagen IV deposition. ESKD was defined as an estimated glomerular filtration rate < 15 ml/min per 1.73 m^2^ or the initiation of renal replacement therapy. A positive family history was defined as female family members with hematuria and/or male family members with chronic kidney disease and/or deafness.

### Genetic Analysis

Genetic analysis was performed at the Laboratory of Clinical Genetics at the Maastricht University Medical Center+, the Netherlands. Genomic DNA was extracted from peripheral blood mononuclear cells and purified using standard DNA extraction methods.

In all participants, regular genetic analyses of the exons and direct flanking regions of the *COL4A3/A4/A5* genes had been performed in the past using either Sanger sequencing or next-generation sequencing based techniques (presented in the following section), both combined with MLPA.

### Alport Panel Sequencing

All coding exons and immediate flanking intron regions (with a region of interest at −40 and +20 base pairs flanking the exon) of *COL4A3*, *COL4A4,* and *COL4A5* (NCBI RefSeq *COL4A3* NM_000091.4, *COL4A4* NM_000092.4, *COL4A5* NM_033380.2) were analyzed using single-molecule molecular inversion probes enrichment and next-generation sequencing for single nucleotide variants and copy number variants detection.

**MLPA** Exon duplication or deletion was performed using MLPA (MLPA kits: *COL4A5*: P191-B3 and P192-B3; *COL4A3*: P439-B1; *COL4A4*: P444-A1; Microbiology Research Centre-Holland, Amsterdam, the Netherlands).

### Variant Interpretation

The pathogenicity of detected DNA variants was assessed using the standards and guidelines of the American College of Medical Genetics and Genomics.^27^ Alamut Visual Plus (version v1.8.1, 2023 SOPHiA Genetics, Rolle, Switzerland), a software tool integrating predictive splicing programs, pathogenicity prediction programs, and multiple online databases, was used for variant interpretation. To determine whether a variant has already been reported before, we used our Alport population as a reference (> 500 patients) and consulted several online databases as follows: Human Gene Mutation Database (Professional 2022.1), Leiden Open Variant Database (v.3.0), and ClinVar database.

### Aberrant Splicing: mRNA Analysis

In addition to regular genetic analysis focusing on variants in the exons of *COL4A3/A4/A5*, we used mRNA analysis to detect abnormal splicing, guiding the search for the causative (deep) intronic variant in cases without causal variants in the exon screenings, or providing confirmation of previously unclassified variants. From all patients, we cultured fibroblasts, and in 1 patient, we used urine derived podocyte-lineage cells to extract mRNA. When abnormal *COL4A5* transcripts were detected and Sanger sequenced, the corresponding aberrant spliced exons and flanking intronic sequences were used to infer the genomic location for further Sanger sequencing to identify the intronic variant causing the aberrant splicing.

### Fibroblast Cell Culture

A patient’s 3 mm skin biopsy was taken and cut into small pieces immersed in Dulbecco's Modified Eagle Medium with 10% fetal calf serum (FCS, Gibco, Thermo Fisher Scientific, Waltham, MA). Fibroblasts were cultivated in 25 cm^2^ culture flasks and grown to confluence.

### Urine Sample Collection and Cell Culture

A fresh urine sample was collected, aliquoted in 50 ml tubes, and kept at 4 °C until further processing. The urine sample was processed within 4 hours after collection. Subsequent cell culture of urine-derived cells was based on an adapted protocol of Zhou *et al.*^28^

In short, the urine sample was centrifuged in 50 ml tubes (Greiner Bio-One, Kremsmünster, Austria) for 10 min, 400 g at room temperature. The supernatant was fully aspirated, and the cell pellet was washed with 10 ml washing buffer consisting of phosphate buffered saline (Gibco) and 1% (v/v) antibiotic-antimycotic (Gibco). Cells were centrifuged again for 10 min, 400 g at room temperature, and the supernatant was fully removed. Cells were reconstituted in DMEM-HAM’s F12 (Gibco) supplemented with 5 μg/ml insulin, 5 μg/ml transferrin, and 5 ng/ml selenium (ITS, Sigma-Aldrich, St. Louis, MO), 10% fetal calf serum (FCS, Gibco), 1% (v/v) penicillin/streptomycin (Gibco) and 0.5% (v/v) amphotericin B/gentamicin (Gibco). Cells were seeded on 1% (w/v) gelatin-coated (Sigma) 6-well cell culture plates (Greiner) and cultured at 37 °C, 5% (v/v) CO_2_. For the expansion of urine-derived cells, the culture medium was refreshed at 3 days after initial seeding to allow for cell attachment. Subsequently, the medium was refreshed every other day until clonal expansion reached 70% to 80% confluency.

The urine-derived cells expressed *COL4A3*, *COL4A4* and *COL4A5* thus confirming the presence of podocyte-lineage cells (Supplementary Methods and Supplementary Figure S1).

### mRNA Isolation, cDNA Synthesis, and COL4A5 cDNA Analysis

For RNA isolation, cells were harvested and processed using the Qiagen RNeasy Mini kit (QIAGEN, Venlo, the Netherlands) according to the manufacturer’s protocol. RNA samples were stored at −80 °C for further processing. cDNA was synthesized with random hexamer primers using the First Strand cDNA Synthesis Kit for Reverse Transcription Polymerase Chain Reaction (Sigma-Aldrich) with instructions provided by the manufacturer. The resulting cDNA was subsequently amplified in a PCR reaction with gene-specific primers listed in Supplementary Table S1. Primers for *COL4A5* cDNA analysis were used as described previously and are listed in Supplementary Table S2.^29^

### Genomic DNA Analysis

Intronic variants were retrospectively deduced from the cryptic mRNA transcripts and genomic DNA fragments amplified with Polymerase Chain Reaction (primers in Supplementary Table S3) and subsequently Sanger sequenced on an ABI Prism 3730XL Genetic Analyzer (Applied Biosystems, Thermo Fisher Scientific) with the BigDye Terminator (v3.1 Cycle Sequencing Kit, Thermo Fisher Scientific).

### In-silico Splicing Analysis

Alamut was used, integrating 4 splice prediction tools (SpliceSiteFinder-like, MaxEntScan, Neural Network Splice, GeneSplicer), a branch-point predictor, and exonic splicing enhancers predictions (ESEfinder, RESCUE-ESE). SpliceAI (Broad Institute, Cambridge, MA) for each variant was also evaluated.

## Results

### Patient Characteristics

We studied 11 unrelated male patients with clinical features of XLAS, with abnormal staining of the collagen-α5 chain on skin biopsies, in whom routine genetic analysis of at least the exons and direct flanking regions of the *COL4A5* gene failed to identify a pathogenic variant (Figure 1). In 9 out of 11 patients, we were able to establish a genetic diagnosis of XLAS using mRNA analysis. All 9 identified variants have not been reported in the literature or databases before.

In 2 patients, mRNA analysis was used as a confirmatory tool to demonstrate pathogenicity. In 2 patients (patients 2 and 3), routine genetic analysis identified a VUS in the direct intronic flanking regions (c.1587+5G>A and c.439-6T>G). In the remaining 9 patients, routine genetic analysis failed to identify a variant, and mRNA analysis was used to screen for aberrant mRNA splicing. Aberrant splicing was detected in 7 patients, and by guiding DNA sequencing allowed identification of a deep-intronic variant. In 2 patients, no aberrant mRNA splicing was detected, thus failing to establish a genetic diagnosis of XLAS.

Table 1 illustrates the clinical characteristics of the 9 patients with a detected pathogenic variant. All patients had initial symptoms during childhood. Six patients developed ESKD, with age at ESKD ranging from 18 to 60 years. Six patients had hearing loss. The patients without a detected variant are young males (10 and 17 years old) with hematuria, normal kidney function, and no hearing loss. Their mothers have microscopic hematuria.

**Table 1:** Clinical characteristics, identified variants, effects, and consequences

ID	Presenting symptoms (age, yr)	CKD stage (age) or ESKD (age)	Hearing loss (yes/no)	Family history (yes/no)	Exon/intron	Nucleotide change	RNA consequence	Aberrant splicing	Amino acid change	Type of variant	Predicted effect	cDNA consequence
1	Hematuria + proteinurie (4)	ESKD (18)	No	Yes	i28	c.2245-41del	r.2245_2395del	Complete	p.(Gly749Valfs∗20)	Frameshift	Branch site loss	Exon 29 skipping
2	Hematuria (0)	G1A3 (20)	Yes	Yes	i23	c.1587+5G>A	r.1517_1587del	Complete	p.(Ser507Hisfs∗14)	Frameshift	Donor splice site loss	Exon 23 skipping
3	Hematuria + proteinuria +decreased kidney function (11)	ESKD (44)	No	Yes	i7	c.439-6T>G	r.438_439ins439-5_439-1	Partial	p.(Gly147Asnfs∗10)	Frameshift	Cryptic acceptor splice site within intron 7	5 bp intron retention
4	Hematuria (1)	ESKD (60)	Yes	Yes	i21	c.1423+1175G>T	r.1423_1424ins1423+1187_1423+1230	Partial	p.(Asp476Profs∗96)	Frameshift	Cryptic acceptor splice site within intron 21	Exon-cassette (44 bp)
5	Hematuria + proteinuria +decreased kidney function (12)	ESKD (19)	Yes	Yes	i30	c.2510-1510A>G	r.2509_2510ins2510-1509_2510-1437	Complete	p.(Gly837Alafs∗11)	Frameshift	Cryptic acceptor splice site within intron 30	Exon-cassette (73 bp)
6	Hematuria (2)	ESKD (20)	Yes	No	i31	c.2677+423C>G	r.2677_2678ins2677+259_2677+418	Complete	p.(Gly893Aspfs∗29)	Frameshift	Cryptic donor splice site within intron 31	Exon-cassette (160 bp)
7	Hematuria +proteinuria (10)	ESKD (27)	Yes	Yes	i6	c.385-673T>G	r.384_385ins385-764_385-678	Complete	p.(Lys128_Gly129ins29)	In-frame insertion	Cryptic donor splice site within intron 6	Exon-cassette (87 bp)
8	Hematuria (16)	G2A3 (43)	No	No	i4	c.277-581A>G	r.276_277ins277-595_277-538	Partial	p.(Pro94Ilefs∗7)	Frameshift	Splicing inhibitor/enhancer sequence within intron 4	Exon-cassette (58 bp)
9	Hematuria +proteinuria (4)	G3bA3 (37)	Yes	No	i6	c.385-707G>A	r.384_385ins385-764_385-618	Partial	p.(Gly129Alafs∗38)	Frameshift	Splicing inhibitor/enhancer sequence within intron 6	Exon-cassette (147 bp)

bp, base pair; CKD, chronic kidney disease; ESKD, end-stage kidney disease; e, exon; I, intron; α5: type IV collagen alpha-5 chain.

Nucleotide changes were named following the Human Genome Variation Society (HGVS) guidelines (https://varnomen.hgvs.org/). For genomic DNA and RNA positioning numbering was based on the reference sequences (NCBI RefSeq GRCh37/hg19 COL4A5: NM_033380.2).

### Splicing Effects

**Figure 2:**
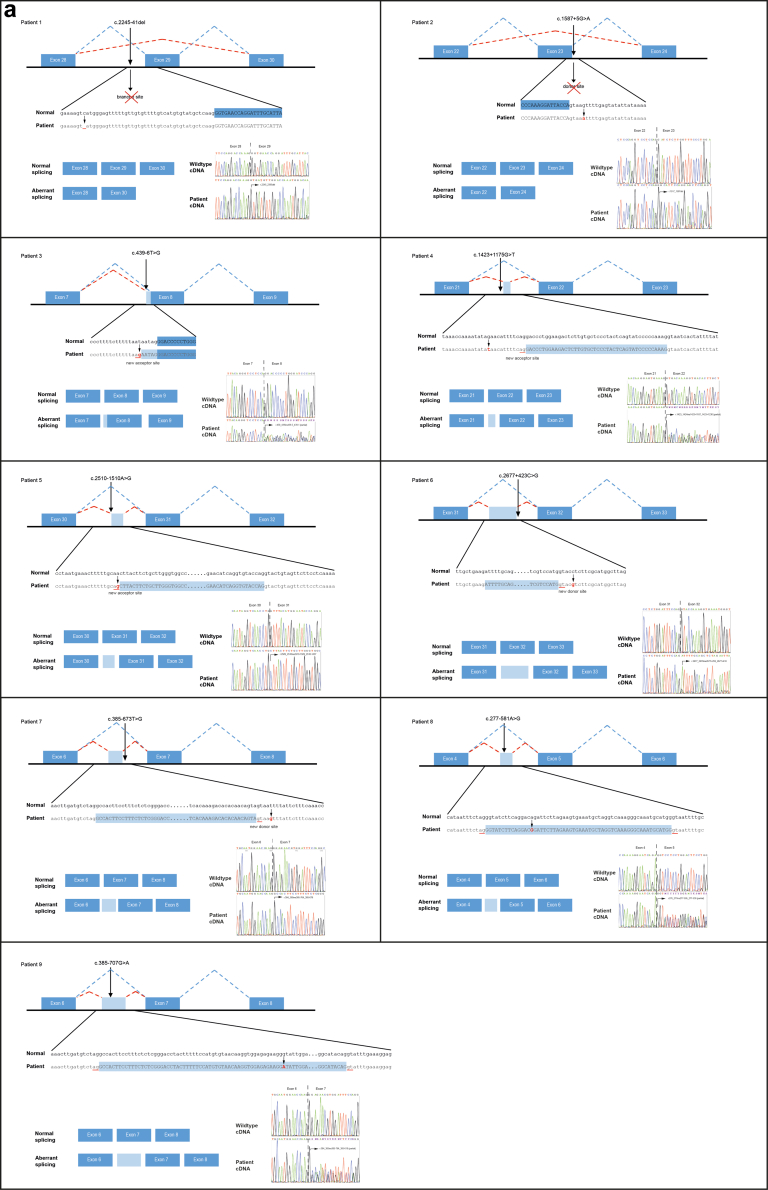

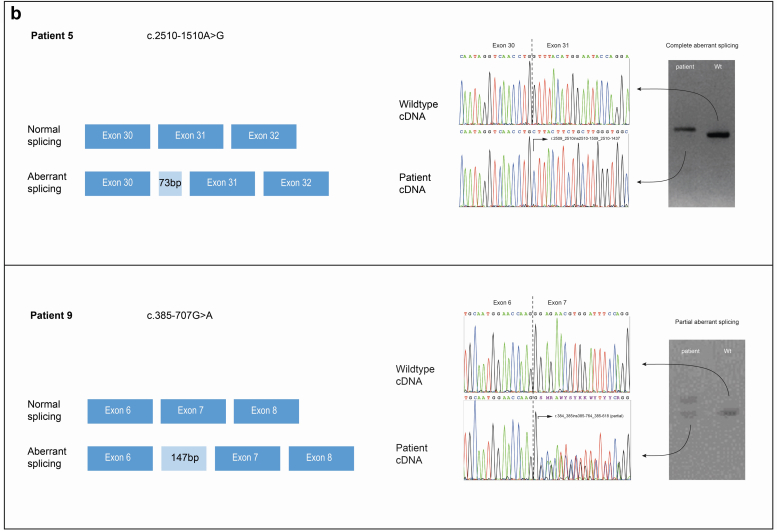
(a) Schematic representation of the aberrant splicing events detected in patients. The upper panels show schematics of splicing with aberrant splicing indicated by red dashed lines and normal splicing by blue lines. The splicing variant is displayed in this schematic. The original nucleotide sequences are shown below with the nucleotide change in the patients’ sequence in red. Inserted sequences are shown by light-blue box in the patient’s sequence, whereas exonic sequences are indicated by blue background in the normal sequence. In the lower panel on the left, the normal (top) and aberrant splicing (below) is schematically shown with exon-cassettes represented as light-blue filled rectangles (with the boxed sequence above in the same color). On the right the electropherograms are shown of the wild-type cDNA sequence (upper panel) and the patient’s cDNA sequence (lower panel). Patient 1: the variant c.2245-41del leads to exon 29 skipping because of a putative loss of a branch site within intron 28; Patient 2: the variant c.1587+5G>A resulted in exon 23 skipping because of loss of a donor splice site within intron 23; Patient 3: the intronic variant c.439-6T>G creates a stronger cryptic acceptor splice site within intron 7 than the native acceptor splice site, resulting in a 5 bp intron retention adjacent to exon 8. The electropherogram of the patient’s cDNA sequence indicates partial aberrant splicing together with normal transcript; Patient 4, the deep-intronic variant c.1423+1175G>T within intron 21 creates a cryptic acceptor splice site within the affected intron and the activation of an existing (weak) donor splice site, resulting in a 44 bp exon-cassette between exon 21 and 22. The electropherogram of the patient’s cDNA sequence indicates partial aberrant splicing together with normal transcript; Patient 5: the deep-intronic variant c.2510-1510A>G within intron 30 creates a cryptic acceptor splice site and the activation of an existing (weak) donor splice site, resulting in a 73 bp exon-cassette between exon 30 and 31; Patient 6: the variant c.2677+423C>G in intron 31 generated a cryptic donor splice site with an existing native (weak) acceptor site, resulting in a 160 bp exon-cassette between exon 31 and 32; Patient 7: the variant c.385-673T>G in intron 6 created a cryptic donor site resulting in the insertion of an exon-cassette (87 bp) between exons 6 and 7; Patient 8, the variant c.277-581A>G probably resides in a splicing inhibitor/enhancer sequence within intron 4, activating local native splice sites and leading to insertion of an exon-cassette (58 bp) between exons 4 and 5. The electropherogram of the patient’s cDNA sequence indicates partial aberrant splicing together with normal transcript; Patient 9: the variant c.385-707G>A resides within a splicing inhibitor/enhancer sequence in intron 6, resulting in a 147 bp exon-cassette between exons 6 and 7. The electropherogram of the patient’s cDNA sequence indicates partial aberrant splicing together with normal transcript. (b) Agarose gel images of complete and partial aberrant spliced PCR-products. On the left, the normal (top) and aberrant splicing (below) is schematically shown with exon-cassettes represented as light-blue filled rectangles. On the right, the electropherograms are shown of the wild-type cDNA sequence (upper panel) and the patient’s cDNA sequence (lower panel). Next to the electropherograms, a representative agarose gel image of the PCR-products sequenced are shown. Patient 5: The variant c.2510-1510A> results in a 73 bp exon-cassette between exon 30 and 31. The agarose gel image of the PCR-products sequenced are shown of complete aberrant splicing of the patient (left) and a normal control (right). Patient 9: The variant c.385-707G>A results in a 147 bp exon-cassette between exons 6 and 7. A representative agarose gel image of the PCR-products sequenced are shown of partial aberrant splicing of the patient (left) and a normal control (right). PCR, polymerase chain reaction.

An overview of the identified variants, including their splicing effects and consequence is also provided in Table 1. The pattern of aberrant splicing is shown in Figure 2a.

In 2 patients (patients 1 and 2), the intronic variants lead to exon skipping. Both variants result in a truncated protein and/or partial nonsense-medicated mRNA decay (NMD), a cellular mechanism that degrades transcripts containing premature stop codons, thereby reducing aberrant transcript levels and contributing to decreased overall *COL4A5* protein expression. In patient 3, the intronic variant results in a 5 base pairs intron retention. In 6 patients (patients 4–9), the deep-intronic variants resulted in the insertion of an exon-cassette derived from an intron between the adjacent exons. The intronic variant identified in patient 9 (c.385-707G>A) is comparable to an earlier reported nucleotide change in intron 6, resulting in an exon-cassette insertion through the same mechanism.^30^

At the translation level, all these variants are predicted to result in a frameshift, leading to a premature stop codon and truncated protein, except for 1 resulting in an in-frame insertion (patient 7).

### Splicing and Phenotype

We found complete aberrant splicing in 5 patients (patients 1, 2, and 5–7) and partial aberrant splicing in the remaining 4 patients (patients 3, 4, 8, and 9), as illustrated in Figure 2b. In the 4 cases with partial aberrant splicing, both the original splice site and the cryptic splice site are used, indicating that the cryptic acceptor splice site or enhancer sequence is probably weak and not used in every splice event. We observed a difference in the severity of the phenotype between patients with complete and partial aberrant splicing (see Table 1). Four of the 5 patients with complete aberrant splicing reached ESKD, compared with 2 or 4 patients with partial aberrant splicing. Also, the age at ESKD tended to be lower in patients with complete aberrant splicing (median 20 years, range: 18–27 years) compared with patients with partial aberrant splicing (median 52 years, range: 44–60 years). We suggest that partial aberrant splicing may also explain the large variation in clinical phenotype within a family, as illustrated by the family of patient 4 (Supplementary Figure S2).

### Urinary Cell-Derived Culture to Evaluate Aberrant mRNA Splicing

The partial cryptic splicing observed by mRNA analysis of urine-derived podocyte-lineage cells from patient 4 was confirmed in a cousin (Supplemental Figure S2; IV-5) using fibroblasts, showing a comparable pattern of partial cryptic splicing. These results suggest that both urine-derived podocyte-lineage cells and fibroblasts provide representative material for mRNA analysis.

## Discussion

This study illustrates the important role of mRNA analysis in the evaluation of patients with suspected XLAS. First, mRNA analysis allows to prove the pathogenicity of variants of unknown significance detected in the immediate flanking intronic regions just outside the exons (-40, +20 base pairs). Secondly, mRNA analysis allows for the detection of aberrant mRNA splicing, guiding subsequent genomic sequencing to identify pathogenic deep-intronic variants in patients with negative test results on conventional diagnostic techniques. Lastly, our data suggest that urine-derived podocyte-lineage cells can be used as a noninvasive procedure for mRNA isolation.

Using these techniques, we were able to establish a genetically proven diagnosis of XLAS in 9 out of 11 patients with clinically suspected XLAS. Specifically, we confirmed the pathogenicity of 2 novel variants identified in routine genetic analysis and discovered 7 novel deep-intronic variants. Only 2 cases remain unresolved. Notably, this high detection rate may reflect patient selection. Our study included patients with suspected XLAS, with a negative genetic diagnosis with routine testing, who responded to our invitation to visit the hospital for additional skin biopsy and cell culture, and consented for this study. Since for privacy reasons there is no information on nonresponders, we can only estimate an overall diagnosis rate in our cohort. Over a 20-year period, a pathogenic variant was found in approximately 200 patients with clinically suspected XLAS (roughly 10 per year). It is estimated that in 10% to 20% of patients with XLAS initial genetic testing is negative, amounting to 20 to 40 patients. Based on these numbers (shown in Figure 1), we estimate that the likelihood of identifying a genetic diagnosis in patients with XLAS and initial negative genetic testing is at least 20% (7 out of ∼ 40). It is likely that the suspicion of XLAS was less in nonresponders (explaining their lack of interest in further studies), and that true discovery rate is higher (maximum 80%). Still, even at 20%, it is clinically relevant to perform additional studies in patients with initially negative genetic results.

Our findings also support the role of incomplete aberrant splicing in causing disease variability and extends on the data presented in the literature data. Horinouchi *et al.*^31^ reported 3 cases with a variant in an exon of *COL4A5* causing abnormal splicing, in whom the disease severity was milder when normal *COL4A5* transcripts persisted. A similar conclusion related to an intronic variant was acknowledged by Nozu *et al.*,^19^ who described an individual with a pathogenic variant in intron 25 (c.1948+894C>G, described as IVS25+894C>G) with both normal and abnormal transcripts in the kidney. This male patient had an extremely mild phenotype. Malone *et al.*^18^ reported a family with XLAS with variation of disease severity, diagnosed with a variant in intron 24 of the *COL4A5* gene (c.1780-6T>G), leading to incomplete abnormal splicing. Recently, Boisson *et al.*^12^ reported 12 male patients with XLAS, caused by deep-intronic variants in the *COLA5* gene, with quantitative measurement of aberrant and total transcript through mRNA analysis of fibroblasts. A correlation was found between the ratio of aberrant transcripts and age at ESKD.^12^ Based on these data, we postulate that the variability of disease severity in our study may be explained by partial aberrant splicing. Admittedly, our data do not allow firm conclusions. Information on complete or partial splicing is based on a method that is semiquantitative at best. Also, we did not use NMD inhibitors in the assay. To proof a strong association between the ratio of abnormal/total transcript new studies are needed, which require renewed patient consent, a skin biopsy, developing a quantitative method, and using this method in the absence and presence of a strong NMD inhibitor. Ideally, such studies should first focus on families which show large interindividual heterogeneity.

In addition, it is important to acknowledge that the amount of normal transcript can vary between cell types, especially in leukocytes and hair roots, compared with fibroblasts or urine-derived podocyte-lineage cells. This is illustrated by Nozu *et al.*,^19^ who reported the presence of normal and abnormal transcripts in the kidney, whereas other evaluated cells (leucocytes and hair root) showed only the abnormal transcripts.

In the upcoming years, an increase in detected intronic VUS can be expected because of the implementation of whole-genome sequencing. This will necessitate the need for reliable functional assays. A reliable functional assay is regarded as an important factor in the American College of Medical Genetics and Genomics classification to assess pathogenicity. mRNA studies are superior for interpreting the consequences of a splicing variant. Therefore, a variant can be classified as ‘pathogenic’ instead of ‘likely pathogenic’ or an even lower classification.^5^^,^^27^^,^^32^ The classification will have direct clinical consequences, such as early start of renin-angiotensin-aldosterone system inhibition and counseling of family members. Studying *COL4A5* mRNA from fibroblasts is a well-established assay for analyzing splicing variants. We also applied a more novel technique using urine-derived podocyte-lineage cells. This is not only a commonly available source but also an elegant, noninvasive approach that allows direct mRNA analysis in disease-relevant cells. Disease-relevant cells are especially important to assess incomplete abnormal splicing as an explanation of genotype-phenotype variation in patients, because of the tissue specificity of splicing events. We propose that mRNA analysis from urine-derived podocytes to detect aberrant splicing will facilitate identifying deep-intronic variants through subsequent genomic DNA sequencing, in patients with autosomal dominant and recessive AS, since the *COL4A3/A4* genes are not expressed in fibroblasts. Therefore, we suggest that urine-derived podocyte-lineage cells would be the preferred approach for mRNA analysis of all 3 *COL4A3/A4/A5* genes for the detection of abnormal splicing in patients suspected of AS without a genetic diagnosis based on routine genetic analysis. For *COL4A5,* however, the expression of partial cryptically spliced transcripts appears comparable in urine-derived podocyte-lineage cells and fibroblasts (probably because these representative cells are responsible for depositing *COL4A5* in the basement membrane of the glomerulus and epidermis, respectively^3^^,^^33^). Consequently, fibroblasts can be a good alternative for analysis of the *COL4A5* gene when culture of urine cells is not possible, for instance, in transplanted patients or when there is limited shedding of podocyte-lineage cells in the absence of proteinuria as a marker of GBM membrane damage.^24^

In addition, mRNA analysis can also be applied to confirm deletions detected by MLPA. In our initial cohort, 1 patient was found to harbor a deletion identified by MLPA in mosaic state (at approximately 0.4 peak ratio, i.e. the variant is present in ∼60% of cells), which was consistent with the mosaic collagen-α5 staining pattern in the GBM of the kidney (as described in the Supplementary Case). This finding was confirmed by analysis of mRNA isolated from fibroblasts (Supplementary Figure S3). Similar to partial aberrant splicing, such mosaic variants may contribute to phenotypic variability.

We were unable to identify a causal variant in 2 of the patients, who showed negative *COL4A5* expression in the skin, but normal *COL4A5* expression in fibroblasts. These patients could have aberrant splicing, with the abnormal transcript degraded by NMD and therefore not detectable in our analysis. Future RNA studies in these patients could include fibroblasts cultured under NMD inhibitors, like emetine.^12^

In summary, this study reports 9 novel pathogenic (deep-) intronic variants in 9 out of 11 patients with clinically suspected XLAS, without a proven genetic diagnosis based on routine genetic analysis. mRNA analysis demonstrating aberrant splicing in particular enabled the identification of pathogenic (deep-) intronic variants by guiding targeted genomic sequencing. This emphasizes the importance of further analysis of noncoding regions when the phenotype strongly suggests AS. There was a striking variation in the age at ESKD, which may be the result of the presence of abnormal and normal *COL4A5* transcripts. Pathogenicity was not only confirmed by mRNA analysis of fibroblasts but also by a noninvasive method for mRNA study of disease-specific cells using urine-derived podocyte-lineage cells. This approach provides an alternative diagnostic method for patients suspected of XLAS as well as autosomal (recessive or dominant) AS. It is applicable when routine genetic analysis of the coding and flanking regions of the *COL4A3*, *COL4A4*, and *COL4A5* genes fails to establish a definitive genetic diagnosis or when there is a need to establish the pathogenicity of an identified variant.

## Disclosure

All the authors declared no competing interests.

## Supplementary Material

Supplementary File (PDF) **Supplementary Methods. Supplementary References. Supplementary Case: Mosaicism. Figure S1.** Podocyte marker detection on urine-derived podocyte-lineage cells. **Figure S2.** Family pedigree of patient 4. **Figure S3.** Schematic representation of alternative splicing event. **Table S1.** Primers used for PCR. **Table S2.** List of primers for *COL4A5* cDNA analysis. **Table S3.** List of primers for *COL4A5* genomic DNA analysis.

## Footnotes

Supplementary File (PDF)

**Supplementary Methods.**

**Supplementary References.**

**Supplementary Case: Mosaicism.**
**Figure S1.** Podocyte marker detection on urine-derived podocyte-lineage cells. **Figure S2.** Family pedigree of patient 4. **Figure S3.** Schematic representation of alternative splicing event. **Table S1.** Primers used for PCR. **Table S2.** List of primers for *COL4A5* cDNA analysis. **Table S3.** List of primers for *COL4A5* genomic DNA analysis.
