# Supplementary material for: mRNA Sequencing to Identify Aberrant Splicing in X-linked Alport Syndrome
Source: Kidney Int Rep. 2026 Apr 22;11(7):106553. doi: 10.1016/j.ekir.2026.106553 (PMC13233539; doi:10.1016/j.ekir.2026.106553)
Supplement: Supplementary File (PDF) — Supplementary Methods. Supplementary References. Supplementary Case: Mosaicism. Figure S1. Podocyte marker detection on urine-derived podocyte-lineage cells. Figure S2. Family pedigree of patient 4. Figure S3. Schematic representation of alternative splicing event. Table S1. Primers used for PCR. Table S2. List of primers for COL4A5 cDNA analysis. Table S3. List of primers for COL4A5 genomic DNA analysis. [file mmc1.pdf]

Supplementary File

Page 2: Supplementary Methods including Table S1 and Figure S1

Page 4: Table S2: List of primers for *COL4A5* cDNA analysis

Page 5: Table S3: List of primers for *COL4A5* genomic DNA analysis

Page 6: Figure S2: Family pedigree of patient 4

Page 7: Supplementary Case: Mosaicism

Page 8: Figure S3: Schematic representation of alternative splicing event

Page 8: Supplementary References

Supplementary Methods: Podocyte marker detection on urine-derived podocyte-lineage cells.

Cells were harvested and RNA was isolated according to the manufacturer's protocol of the RNeasy kit (Qiagen, #74104). Next, RNA concentrations and quality were determined by using the Nanodrop 1000 system (ThermoFisher). Total RNA (100 ng) was reverse transcribed into first-strand cDNA according to the manufacturer's protocol of the GoScript reverse transcriptase kit (Promega, #A5001). cDNA was subsequently amplified in a PCR reaction using the AmpliTaq gold 360 kit (ThermoFisher, #4398881). The primers used were generated using the Primer-BLAST tool (National library of Medicine, USA) (Table S1), all exon-exon spanning to limit detection of genomic DNA and expected product size was validated using human kidney cortex cDNA. All PCR products were loaded on a 1% Agarose gel, and imaged using the Proxima gel and blot imaging platform (Isogen Life Science) (Figure S1).

Table S1. Primers used for PCR.

Gene	Strand	Sequence	Product size
COL4A3	Forward	GACTCGCCCAGGCTCTGA	280
	Reverse	AAGTCCTGGAAAGCCCTTGG	
COL4A4	Forward	GGTCCAACTGGTGTTCCTGG	218
	Reverse	ACGGCACCTAAAATGAACAC	
COL4A5	Forward	GCTATGGGTGTTCTCCAGG	227
	Reverse	GGTGTCCCTGGAAATCCAG	
NPHS1	Forward	TAGGCAGTTGCTGGTCTGTG	393
	Reverse	GCACTAGGGGGAAAGGTGAC	
NPHS2	Forward	CATCTGGTTCTGCGTAAAGGTTG	381
	Reverse	ACCTTTGCATCTTGGGCGAT	
SYNPO	Forward	GAGGACCTAGCAGACGTTGG	456
	Reverse	AGCAGTGCTGCATTCTCCTT	
PODXL	Forward	AGAAGCAGCTCGTCCTGAAC	205
	Reverse	TTGGCAGGGAGCTTAGTGTG	
WT1	Forward	GTTACAGCACGGTCACCTTC	143
	Reverse	CACCGAGTACTGCTGCTCAC	
PAX2	Forward	CTTTCCACCCAACGCCGGAT	239
	Reverse	CCGCAAACTGTCCACACCAC	
PAX8	Forward	CCAGCTCAGCTGTAACTCCC	267
	Reverse	CATAGGCCTCTGGGTAGTGC	
ACTB	Forward	CTTCGCGGGCGACGAT	104
	Reverse	CCACATAGGAATCCTTCTGACC	

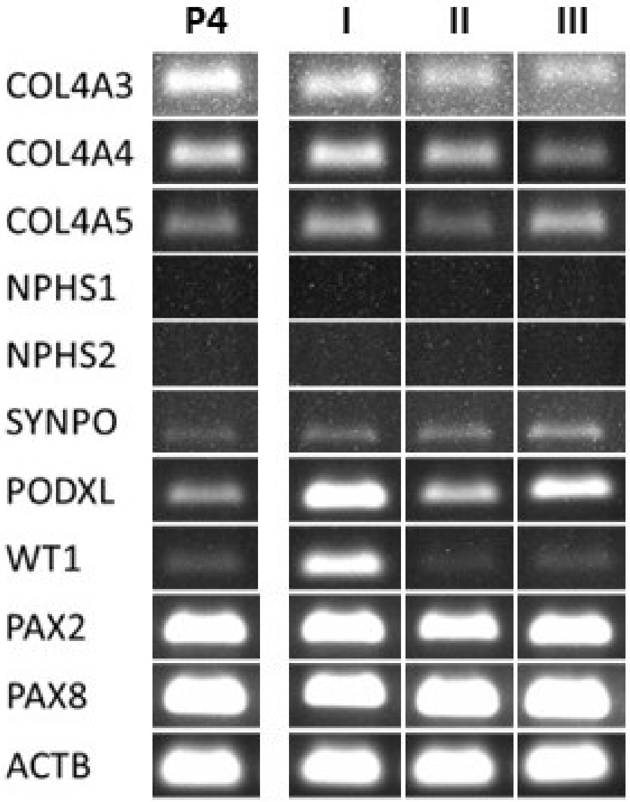

Figure S1 Podocyte marker detection on urine-derived podocyte-lineage cells.

PCR analysis of urine- derived podocyte lineage cells of patient 4 (P4) and three reference XLAS patients: I= female, intronic *COL4A5* variant, II= male, exonic *COL4A5* variant, III= male, intronic *COL4A5* variant.
Urine- derived podocyte lineage cells showed expression of the podocyte-specific markers *COL4A3/A4/A5* and synaptopodin, dedifferentiation markers (PAX2, PAX8), and lack expression of NPHS1 (nephrin) and NPHS2 (podocin).

Abbreviations: XLAS: X-linked Alport Syndrome; NPHS1: nephrosis 1 (nephrin); NPHS2: nephrosis 2 (podocin); SYNPO: synaptopodin; PODXL: podocalyxin; WT1: Wilms Tumor 1; PAX2: Paired Box Gene 2; PAX8: Paired Box Gene 8; ACTB: actin beta

Table S2: COL4A5 cDNA PCR Primer List

Primer name	Sequence	Product length (bp)
COL4A5_01_RNA_F	CTGCCGGCTTGTTCTTACTG	413
COL4A5_07_RNA_R	TGGAGGACCCTGTAAACCAG	413
COL4A5_04_RNA_F	CCAGGACCAAAAGGAATCAG	420
COL4A5_12_RNA_R	GGGTCCCTGGAAATTTAAGC	420
COL4A5_11_RNA_F	CCAGGACTTCCAGGACCTAA	503
COL4A5_19_RNA_R	GGAAATCCTCGCTCTCCTTT	503
COL4A5_16_RNA_F	ACCAGGCAAAGATGGAGAAA	562
COL4A5_22_RNA_R	TCCAATGCAGTTGAAGCAAG	562
COL4A5_19_RNA_F	CTGGAGAAAAAGGAGAGCGA	470
COL4A5_23_RNA_R	AGTTGCACCAGCTTGTCCTT	470
COL4A5_21_RNA_F	TCCAAGGAGAACAAGGAGTGA	588
COL4A5_26_RNA_R	CCGGCTGGGTTATAGTCTGA	588
COL4A5_22_RNA_F	TTCAACTGCATTGGAACTGG	476
COL4A5_25_RNA_R	GCCACACCTTGTATGCCTTT	476
COL4A5_23_RNA_F	TGGTCCCAAAGGATTACCAG	649
COL4A5_28_RNA_R	AGGAGGGCCTTCTAGACCAA	649
COL4A5_25_RNA_F	AGGCCTCCCAGGGAATATAG	504
COL4A5_29_RNA_R	AAACCACGATCACCTTTTGG	504
COL4A5_27_RNA_F	CCTGGTAGCAAAGGAGAACC	458
COL4A5_31_RNA_R	TCCCCCTTCTCTCCTGGTAT	458
COL4A5_28_RNA_F	TGGGACACCTGGAAGAATTG	525
COL4A5_32_RNA_R	CCCATCATACCCATTTCACC	525
COL4A5_29_RNA_F	AGCACTTGGTCCAAAAGGTG	538
COL4A5_33_RNA_R	AAGGCCAGGCTCTCCTTTAC	538
COL4A5_31_RNA_F	CCTCCTGGACTTGATGTTCC	400
COL4A5_34_RNA_R	AACCTTTTGGCCCTGAAACT	400
COL4A5_32_RNA_F	CTGGCAGGAGTGGTGTACCT	504
COL4A5_36_RNA_R	GACCAGGAAGACCTGGAAGA	504
COL4A5_33_RNA_F	GGCCCTACAGGAGAAAAAGG	771
COL4A5_40_RNA_R	GTTTCCAAAGCCTGGTTGAC	771
COL4A5_35_RNA_F	TCCCAAAGGTAACCCTGGTC	535
COL4A5_39_RNA_R	TGGTTCACCCTTCTGTCCA	535
COL4A5_38_RNA_F	AAGGTATTAGTGGCCCTCCTG	522
COL4A5_44_RNA_R	CTCCTTTCAAACCAGGCAAG	522
COL4A5_39_RNA_F	AGCTGGACAGAAGGGTGAAC	560
COL4A5_47_RNA_R	GACCAGGTAATCCAGGAGGA	560
COL4A5_41_RNA_F	TCCAGCTCTGGAAGGACCTA	518
COL4A5_48_RNA_R	CAAAGCCAGGGAGTCCATT	518
COL4A5_47_RNA_F2	GATGCTGGTCCTCCAGGAAT	538
COL4A5_50_RNA_R	TTAGGGGTTGCATGCTCATT	538
COL4A5_47_RNA_F	GCCTGGGCTAAAGGGTCTAC	800
COL4A5_52_RNA_R	CCAGCCAAAAGCTGTAGGAG	800
COL4A5_49_RNA_F	TAAAAGAGCCCACGGTCAAG	515
COL4A5_53_RNA_R	TCAGCGTTTCTGACTGAGGTT	515

bp: base pair

Table S3: COL4A5 gDNA PCR Primer List

Patient ID	Intron	Nucleotide change	Forward Primer sequence	Reverse Primer sequence	Product length (bp)
5	21	c.1423+1175G>T	TGTGTGTGTGTGTGTGTATG	CAAGATTAGGCAAATTAATATATGCCA	158
6	30	c.2510-1510A>G	CTATATGAACATCTAAGCAAATGCCT	GCTCCAAGAGAGCAGTTCTATAA	359
7	31	c.2677+423C>G	CCACTGCAGCCTCAAAC	AGAAGTCTTACAGGCCAGTCA	250
8	6	c.385-673T>G	CTTCCTTTCTCTCGGGACCT	TGAGACGATCACGCCATAAA	495
9	4	c.277-581A>G	TGTTTCTAAACTTTAGCTATAATAATGCTG	TCTCTGTAGGTGAGACTATGGT	231
10	6	c.385-707G>A	CTTCCTTTCTCTCGGGACCT	TGAGACGATCACGCCATAAA	495

bp: base pair

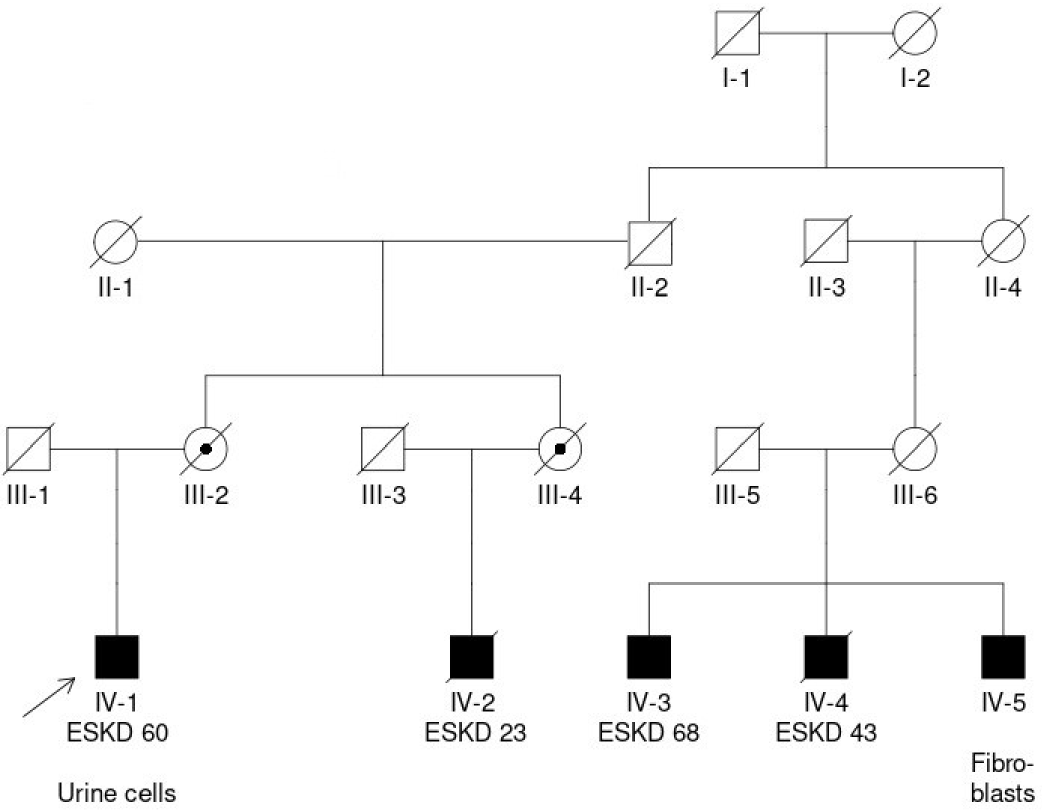

Figure S2 Family pedigree of patient 4

The pedigree illustrates the variability of age at ESKD among male XLAS patients in this family, which ranges from 23 to 68 years. The arrow indicates the index case (patient 4), in whom partial aberrant splicing was detected by mRNA analysis of urine-derived podocyte-lineage cells. A similar pattern of partial cryptic splicing was observed in mRNA analysis using fibroblasts from second-degree cousin IV-5 (aged 62 years), who has not developed ESKD to date.

Non-consenting individuals are not shown in detail. Symbols: square: male; circle: female; black-filled symbols indicate affected male patients with Alport Syndrome; dots indicate female heterozygous carriers with hematuria;
Abbreviations: ESKD: end-stage kidney disease, with age in years.

Supplementary Case: Mosaicism

In our initial cohort we identified a male patient, with a clinical suspicion of XLAS, presenting with hematuria and proteinuria at the age of 3 and hearing loss later in life. CKD stage is G3A3 at the age of 36. Staining of the GBM in a kidney biopsy showed a mosaic pattern of the collagen-α5 chain.
MLPA identified a *COL4A5* exon 37 deletion (c.(3246+1_3247-1)_(3373+1_3374-1)del), a novel variant, in the peripheral blood of a patient in a mosaic state (at approximately 0.4 peak ratio, i.e. the variant is present in ~60% of cells). It was confirmed by performing RT-PCR on cDNA derived from mRNA isolated from fibroblasts, in which only the variant was present (peak ratio 0). The breakpoint of the deletion variant is presumably located within introns 36 and 37 and has not been further characterized on a genomic level (Figure S3).
This exon deletion, present in both mRNA and DNA, results in a frameshift, leading to a premature stop codon (p.(Gly1083Glufs*27)). This probably results in a truncated protein and/or partial NMD. These genetic findings are consistent with the immunohistochemical mosaic staining pattern of the collagen-α5 chain in the epidermal basement membrane (EBM) of the skin and GBM of the kidney in this patient (not shown).
The percentage of mosaicism in kidney tissue and consequently the renal prognosis cannot be directly inferred from blood analysis. However, since the mosaic state is confirmed by the collagen-α5 staining pattern in the kidney biopsy, and although fibroblasts are less representative, the variant was also detected in these cells. Together these findings suggest an early embryonic mosaicism, with the variant present in a substantial fraction of cells across tissues (60%).[S1]

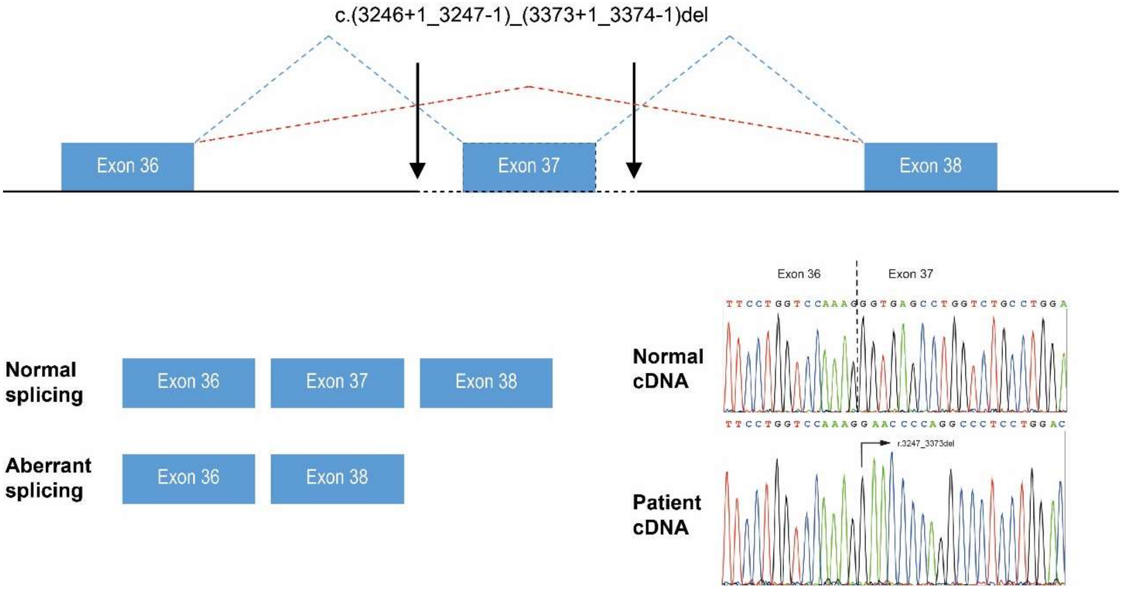

Figure S3 Schematic representation of alternative splicing event

The variant c.(3246+1_3247-1)_(3373+1_3374-1)del leads to a deletion of exon 37.

The upper panel show schematics of splicing with aberrant splicing indicated by red dashed lines and normal splicing by blue lines. The splicing variant is displayed by arrows in this schematic. In the lower panel on the left, the normal (top) and aberrant splicing (below) is schematically shown. On the right the electropherogram is shown of the wild-type cDNA sequence (upper panel) and the patient's cDNA sequence (lower panel) from the cultured fibroblasts (not represented by the mosaicism).

Supplementary references

S1. Kim JH, Hwang S, Son H, et al. Analysis of low-level somatic mosaicism reveals stage and tissue-specific mutational features in human development. *PLoS Genet*. 2022;18(9):e1010404. doi:10.1371/journal.pgen.1010404
